# Role of IGF-Binding Protein 3 in the Resistance of EGFR Mutant Lung Cancer Cells to EGFR-Tyrosine Kinase Inhibitors

**DOI:** 10.1371/journal.pone.0081393

**Published:** 2013-12-05

**Authors:** Yun Jung Choi, Gun Min Park, Jin Kyung Rho, Sun Ye Kim, Gwang Sup So, Hyeong Ryul Kim, Chang-Min Choi, Jae Cheol Lee

**Affiliations:** 1 Department of Pulmonary and Critical Care Medicine, Asan Medical Center, College of Medicine, University of Ulsan, Seoul, Korea; 2 Asan Institute for Life Sciences, Asan Medical Center, College of Medicine, University of Ulsan, Seoul, Korea; 3 Department of Thoracic and Cardiovascular Surgery, Asan Medical Center, College of Medicine, University of Ulsan, Seoul, Korea; 4 Department of Oncology, Asan Medical Center, College of Medicine, University of Ulsan, Seoul, Korea; 5 Department of Internal Medicine, Daehan Hospital, Seoul, Korea; University Magna Graecia, Italy

## Abstract

Most patients treated with EGFR-tyrosine kinase inhibitors (EGFR-TKIs) eventually develop acquired resistance. Loss of expression of insulin-like growth factor (IGF)-binding protein-3 (IGFBP-3) has been suggested as a possible mechanism of resistance to EGFR-TKIs in the A431 and HN11 cell lines. Here, we investigated IGFBP-3 expression in two EGFR mutant lung cancer cell lines with resistance to EGFR-TKIs and examined the value of serum IGFBP-3 level as a marker of resistance. The effect of the induction or suppression of IGFBP-3 expression on resistance was also evaluated. HCC827 sublines with resistance to gefitinib (HCC827/GR) and erlotinib (HCC827/ER) were established. Loss of IGFBP-3 expression was detected by Western blotting in both cell lines without changes in transcriptional activity, and ELISA showed significantly lower amounts of secreted IGFBP-3 in the culture media of the mutant cell lines than in that of the parental line. Despite the loss of IGFBP-3 expression, IGFR signalling activity remained unchanged. Forced expression of IGFBP-3 by adenovirus-mediated transfection or recombinant IGFBP-3 slightly increased the growth-inhibitory and apoptotic effects of EGFR-TKIs, whereas suppression of IGFBP-3 did not affect sensitivity to EGFR-TKI. Serum IGFBP-3 levels measured by ELISA before and after the development of EGFR-TKI resistance in 20 patients showed no significant changes (1815.3±94.6 ng/mL before treatment vs. 1778.9±87.8 ng/mL after EGFR-TKI resistance). In summary, although IGFBP-3 downregulation is associated with the acquisition of resistance to EGFR-TKIs regardless of the mechanism, its effect on resistance was not significant, indicating that IGFBP-3 may not play an important role in resistance to EGFR-TKIs and serum IGFBP-3 level is not a reliable indicator of resistance.

## Introduction

EGFR is a transmembrane receptor that belongs to a family of four related proteins, EGFR (ErbB-1), HER2/neu (ErbB-2), HER3 (ErbB-3) and HER4 (ErbB-4) [1]. Upon ligand binding, EGFR forms homo- or heterodimers with other ErbB receptors leading to the activation of intracellular signalling cascades. The two major intracellular pathways activated by EGFR are the RAS-RAF-MEK-MAPK pathway, which controls gene transcription, cell-cycle progression and cell proliferation, and the PI3K-Akt pathway, which activates a cascade of anti-apoptotic and prosurvival signals [2].

Non-small cell lung cancers (NSCLCs) that harbour activating mutations and/or amplification of the EGFR locus are particularly sensitive to EGFR-tyrosine kinase inhibitors (TKIs) such as gefitinib (Iressa; AstraZeneca International) and erlotinib (Tarceva; OSI Pharmaceuticals) [3]–[9]. Approximately 70–80% of NSCLCs harbouring a somatic mutation in the tyrosine kinase domain of the EGFR gene respond to gefitinib/erlotinib [3], [4], [10]. However, acquired resistance to EGFR-TKI therapy almost always develops after a median of approximately 10 months from the onset of treatment, even in patients who exhibit an initial dramatic response to these agents. Acquired resistance has been associated with a secondary mutation in the EGFR gene, T790M [11], [12], which has been detected in approximately 50% of cancers with acquired resistance to EGFR-TKIs [13], [14]. In addition, amplification of the MET oncogene was identified as another mechanism of acquired resistance mediated by the phosphorylation of ErbB-3 and the consequent activation of PI3K [15], [16]. Similarly, overexpression of the AXL kinase has been associated with resistance to EGFR-TKIs [17].

In a recent study, loss of expression of insulin-like growth factor (IGF)-binding protein 3 (IGFBP-3) was suggested as a possible mechanism of resistance in the A431 and HN11 cell lines [18]. In that study, acquired resistance to EGFR-TKIs was modelled using the A431 squamous cancer cell line, which harbours wild-type EGFR gene amplification. The gefitinib-resistant A431 cell line A431 GR maintained PI3K signalling in the presence of gefitinib by activating the IGF1 receptor (IGF1R) pathway. Inhibition of IGF1R signalling restored the ability of gefitinib to downregulate PI3K/Akt signalling and inhibit A431 GR cell growth. Gene expression analyses showed significant downregulation of IGFBP-3 expression in A431 GR cells, and addition of recombinant IGFBP-3 restored the ability of gefitinib to downregulate PI3K/Akt signalling and to inhibit cell growth. In a different model of acquired gefitinib resistance established in the gefitinib-sensitive wild-type EGFR expressing HN11 head and neck cancer cell line, Akt phosphorylation was maintained in the presence of gefitinib, and resistance was overcome by combined EGFR and IGF1R inhibition. Collectively, these results suggest that loss of expression of IGFBPs in tumour cells treated with EGFR-TKIs results in the activation of IGF1R signalling, which in turn mediates resistance to EGFR antagonists. Therefore, combined therapeutic inhibition of EGFR and IGF1R may abrogate this acquired mechanism of drug resistance. However, a model of acquired resistance to gefitinib has not been developed in EGFR mutant lung cancer cells, which is of clinical importance.

Most of the circulating IGF-1 binds to the principal IGF-binding protein, IGFBP-3 [19]. Serum IGF-1 and IGFBP-3 concentrations can be measured easily and could be of value as indicators of cancer risk. Epidemiological studies have shown that high IGF-1 and low IGFBP-3 levels are independently associated with a high risk of common cancers, including lung cancer [20]. IGFBP-3 has been suggested as a potential target for lung cancer treatment, as adenovirus-mediated overexpression of IGFBP-3 inhibited the growth of NSCLC cells in vitro and in vivo by inducing apoptosis through the inhibition of the PI3K/Akt/PKB and MAPK signalling pathways [21].

In the present study, IGFBP-3 expression was examined in EGFR mutant lung cancer cells with acquired resistance to EGFR-TKIs, and the value of serum IGFBP-3 level was evaluated as a marker of resistance. The effect of induced IGFBP-3 on overcoming resistance was also evaluated.

## Materials and Methods

### Cell Culture and Reagents

The HCC827 cell line was purchased from the American Type Culture Collection (ATCC: Rockville, MD, USA). Cells were cultured in RPMI 1640 medium containing 10% foetal bovine serum (FBS), 2 mM l-glutamine and 100 units/mL penicillin and streptomycin, and maintained at 37°C in a humidified chamber containing 5% CO_2_. Gefitinib and erlotinib were purchase from Selleck Chemicals (Houston, TX, USA). Recombinant human IGFBP-3 (rh IGFBP-3) was purchased from Sigma Aldrich (St. Louis, MO, USA). The adenoviral vector expressing human IGFBP-3 (Ad/IGFBP3) was kindly provided by Dr. Ho-Young Lee (The University of Texas M. D. Anderson Cancer Center) [21].

### Establishment of the Gefitinib- and Erlotinib-resistant Cell Lines

Gefitinib and erlotinib-resistant variants of HCC827 were isolated by stepwise exposure to increasing doses of gefitinib and erlotinib. HCC827 cells were treated with 10 nM gefitinib and erlotinib for 72 h. Cells were continuously exposed to increasing drug concentrations of up to 1 µM over 8 months. EGFR-TKI-resistant colonies were selected at exposure concentrations of 5 µM and the two isolated clones were designated as HCC827/GR and HCC827/ER, respectively. Generation of HCC827/ER cell line has been described previously [17]. Resistant cells were maintained in drug-free medium for at least 2 weeks prior to experiments to eliminate the effects of the drugs.

### Cell Viability Assay

The viability of the cells was measured using the MTT assay and trypan blue cell counting. Cells were plated in 96-well sterile plastic plates and exposed to varying concentrations of drugs in medium containing 1% FBS. After 72 h, 3-(4,5-dimethylthiazol-2-yl)-2,5-diphenyltetrazolium bromide (MTT) solution was added and crystalline formazan was solubilised with sodium dodecyl sulfate (SDS) solution.

The combination effects were evaluated with the trypan blue cell counting. HCC827/GR and HCC827/ER cells were plated in 60 mm dishes and treated with EGFR-TKIs and Ad/IGFBP-3 infection or rh IGFBP-3 for 72 h in medium containing 1% FBS. The cell numbers were determined with an ADAM-MC automatic cell counter (NanoEnTek, Seoul, Korea), according to the manufacturer’s instructions. Results are representative of at least three independent experiments and the error bars signify standard deviations (SDs).

### Western Blot

Proteins were separated on SDS-polyacrylamide gels, and electrotransferred to Immobilon-P membranes (Millipore, Bedford, MA, USA). Antibodies specific for p-EGFR, EGFR, MET, Her2, Her3, IGF1R, p-Akt, Akt, p-ERK, ERK, Axl, IGFBP-3 and actin were obtained from Santa Cruz Biotechnology (Santa Cruz, CA), and those for p-HER2, p-ErbB3, p-MET and p-IGF1R (Tyr1131 and Tyr1135/1136) from Cell Signaling Technology (Beverly, MA). Proteins were detected with an enhanced chemiluminescence Western blotting kit (Amersham Biosciences, NJ, USA), according to the manufacturer’s instructions.

### Semi-quantitative Reverse Transcription-polymerase Chain Reaction (RT-PCR)

Total RNA isolation and cDNA synthesis were performed using the RNA mini-kit protocol (Qiagen Inc., Valencia, CA) and Accupower RT mix reagent, according to the manufacturer’s instructions (Bioneer Corp., Seoul, Korea). The oligonucleotide sequences for amplification were as follows: forward primer, 5′- ATATGGTCCCTGCCGTAGA-3′, and reverse primer, 5′-AAATCGAGGCTGTAGCCAG-3′, for IGFBP-3; forward primer, 5′-GCGAGAAGATGACCCAGATC-3′, and reverse primer, 5′-CCAGTGGTACGGCCAGAGG-3′, for β-actin.

### Transfection of Small Interfering RNA

Small interfering RNA (siRNA) oligonucleotides specific to IGFBP-3 were obtained from Thermo (Thermo Electron Corp., Waltham, MA; IGFBP-3 siRNA-1) and Qiagen (IGFBP-3 siRNA-2). Transfection of siRNA was performed using Lipofectamine 2000 (Invitrogen, Carlsbad, CA) in accordance with the manufacturer’s instructions. Target gene expression was measured 24 h later using western blot analysis. For the MTT assay, cells were seeded onto 96-well plates after siRNA transfection, then treated with the indicated drugs for 72 h.

### ELISA for Serum IGFBP-3 ELISA

Residual serum after routine chemistry test was collected from 20 patients before EGFR-TKI therapy and after acquisition of EGFR-TKIs resistance with written informed consent. It was stored at −80°C until assay. IGFBP-3 concentrations in the cell culture medium and serum were measured using a human IGFBP-3 ELISA kit (R & D Systems, MM, USA) according to the manufacturer’s instructions. Serum samples were diluted to 1∶100 in diluent buffer included in the kit and analysed in duplicate. This assay was performed in a research laboratory of Asan Medical Center and the study protocol was approved by the Institutional Review Board of Asan Medical Center (IRB No; 2010-0267).

### Statistical Analysis

Unless otherwise stated, all values are given as mean ± standard deviation for continuous variables or frequencies and percent for categorical variables. Continuous variables were compared using the Mann-Whitney U test. All data were analyzed using SPSS software (version 12.0, IBM-SPSS, Armonk, NY).

## Results

### Establishment and Characterisation of Gefitinib and Erlotinib-resistant HCC827 Cells with a Deletion Mutation of Exon 19 of the EGFR Gene

**Figure 1:**
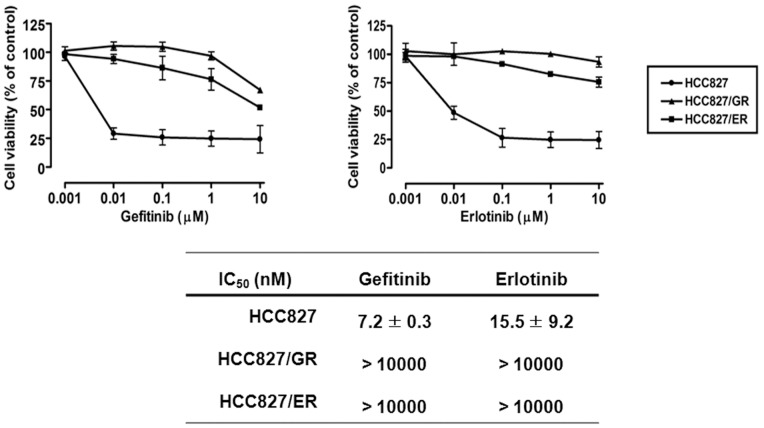
Cytotoxicity and IC_50_ values of EGFR-TKIs in parental HCC827 and resistant cell lines. HCC827, HCC827/GR and HCC827/ER2 cells were treated with the indicated concentrations of gefitinib and erlotinib for 72 h in medium containing 1% FBS. Cell viability and IC_50_ values were determined using the MTT assay.

**Figure 2:**
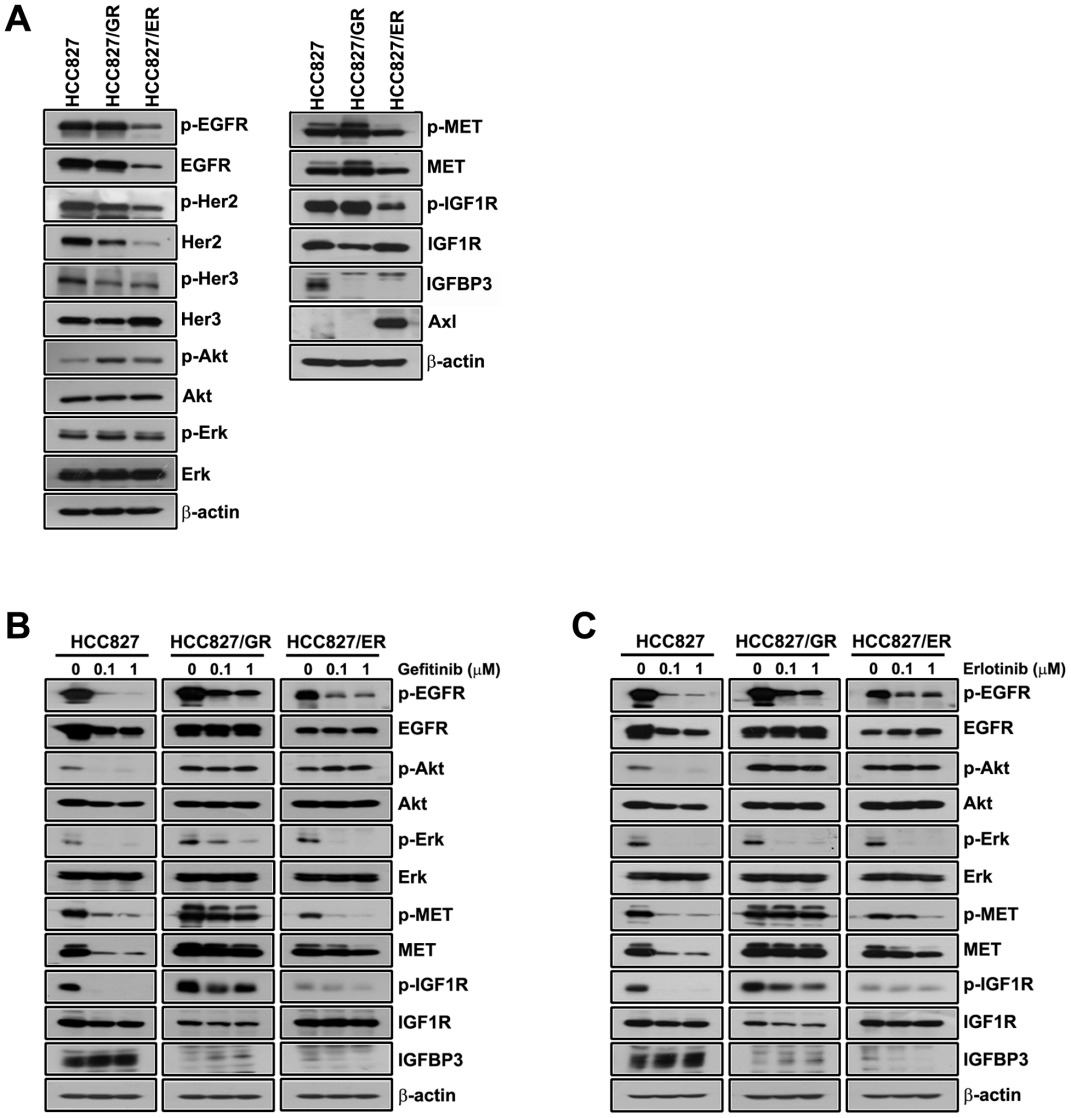
Expression of EGFR-related signals in HCC827 and resistant cell lines. (A) Basal expression of EGFR and EGFR-related signalling molecules in HCC827, HCC827/GR and HCC827/ER cells were evaluated by Western blotting. The effects of gefitinib (B) and erlotinib (C) on EGFR-related signalling were also examined. HCC827, HCC827/GR and HCC827/ER cells were treated with 0.1 and 1 µM gefitinib and erlotinib for 72 h in medium containing 1% FBS. Protein (30 µg) from cell lysates was subjected to Western blot analysis for the indicated proteins.

Two EGFR-TKI-resistant sublines, HCC827/GR and HCC827/ER, were established from parental HCC827 cells by continuous exposure to gefitinib and erlotinib over a period of 8 months. The effects of erlotinib on cell viability were determined with the MTT assay. As shown in Figure 1, the IC_50_ values for gefitinib and erlotinib were 1000-fold higher in the HCC827/GR and HCC827/ER cell lines than in parental HCC827 cells, and the EGFR-TKI-resistant sublines exhibited cross-resistance to each inhibitor. The expression and phosphorylation of EGFR signalling-related proteins involved in sensitivity to EGFR-TKIs were assessed by Western blotting. MET expression and activation were increased in HCC827/GR cells (Figure 2A) harbouring MET gene amplification (data not shown), suggesting that MET amplification contributes to resistance in HCC827/GR cells. This was supported by combination treatment with EGFR-TKI and PHA665752, a MET inhibitor, which effectively suppressed the growth of HCC827/GR cells (data not shown). In HCC827/ER cells, total and activated EGFR were downregulated, and Her2, Her3 and MET levels were significantly lower than those of the parent cells (Figure 2A). However, expression of the Axl kinase was significantly higher in HCC827/ER cells than in the parental line, as reported in our previous study (17). The secondary T790M mutation was not detected in the HCC827/GR and HCC827/ER cell lines by sequencing (data not shown). To assess the inhibitory effects of EGFR-TKIs on MET further, EGFR and their downstream signals, HCC827, HCC827/GR and HCC827/ER cells were treated with gefitinib and erlotinib for 72 h. As shown in Figure 2B and C, the EGFR-TKIs inhibited EGFR phosphorylation in parent and resistant cells but did not suppress MET activation in HCC827/GR, which differed from the other cell lines; downstream Akt signalling was persistently activated in the resistant sublines HCC827/GR and HCC827/ER.

### Loss of IGFBP-3 in HCC827/GR and HCC827/ER Cells

**Figure 3:**
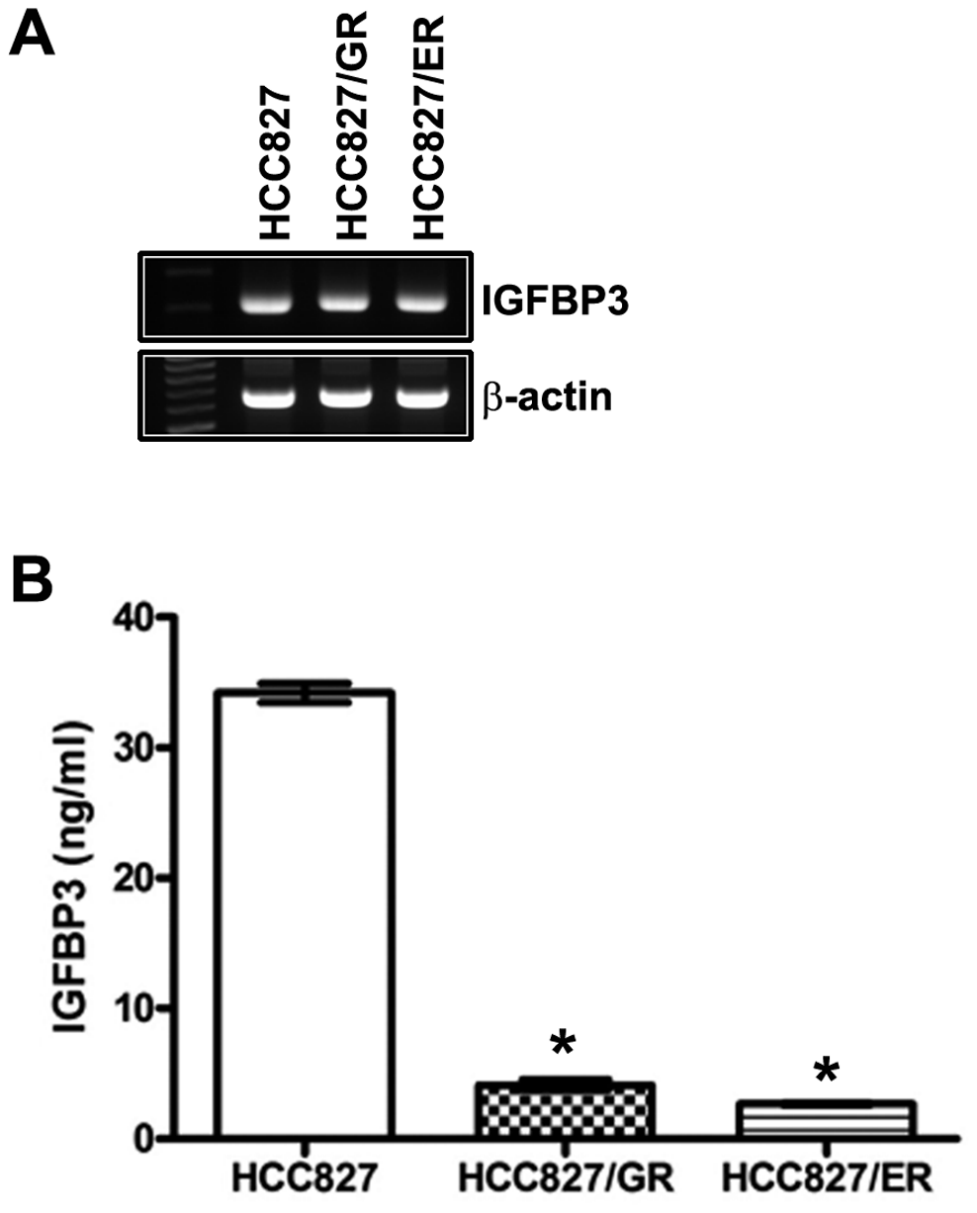
Loss of IGFBP-3 in resistant cell lines and culture medium. IGFBP-3 mRNA (A) and secreted IGFBP-3 (B) were determined by RT-PCR and ELISA in HCC827, HCC827/GR and HCC827/ER. The ELISA was repeated three times and the error bars represent standard deviation (SD). *P<0.001 compared with HCC827 cells.

Assessment of IGFBP-3 expression and IGF1R signalling in parental and resistant cell lines showed that IGFBP-3 expression was significantly downregulated in HCC827/GR and HCC827/ER cells; however, no significant differences in total and activated IGF1R were detected between resistant and parental cells (Figure 2). IGFBP-3 secretion was significantly decreased in HCC827/GR and HCC827/ER cells in parallel with its decreased expression level (Figure 3B). However, no correlation between IGFBP-3 protein and mRNA levels was detected (Figure 3A), suggesting that the downregulation of IGFBP-3 occurs at the post-transcriptional level.

### Sensitivity to EGFR-TKIs in Resistant cells was not Restored by Induction of IGFBP-3

**Figure 4:**
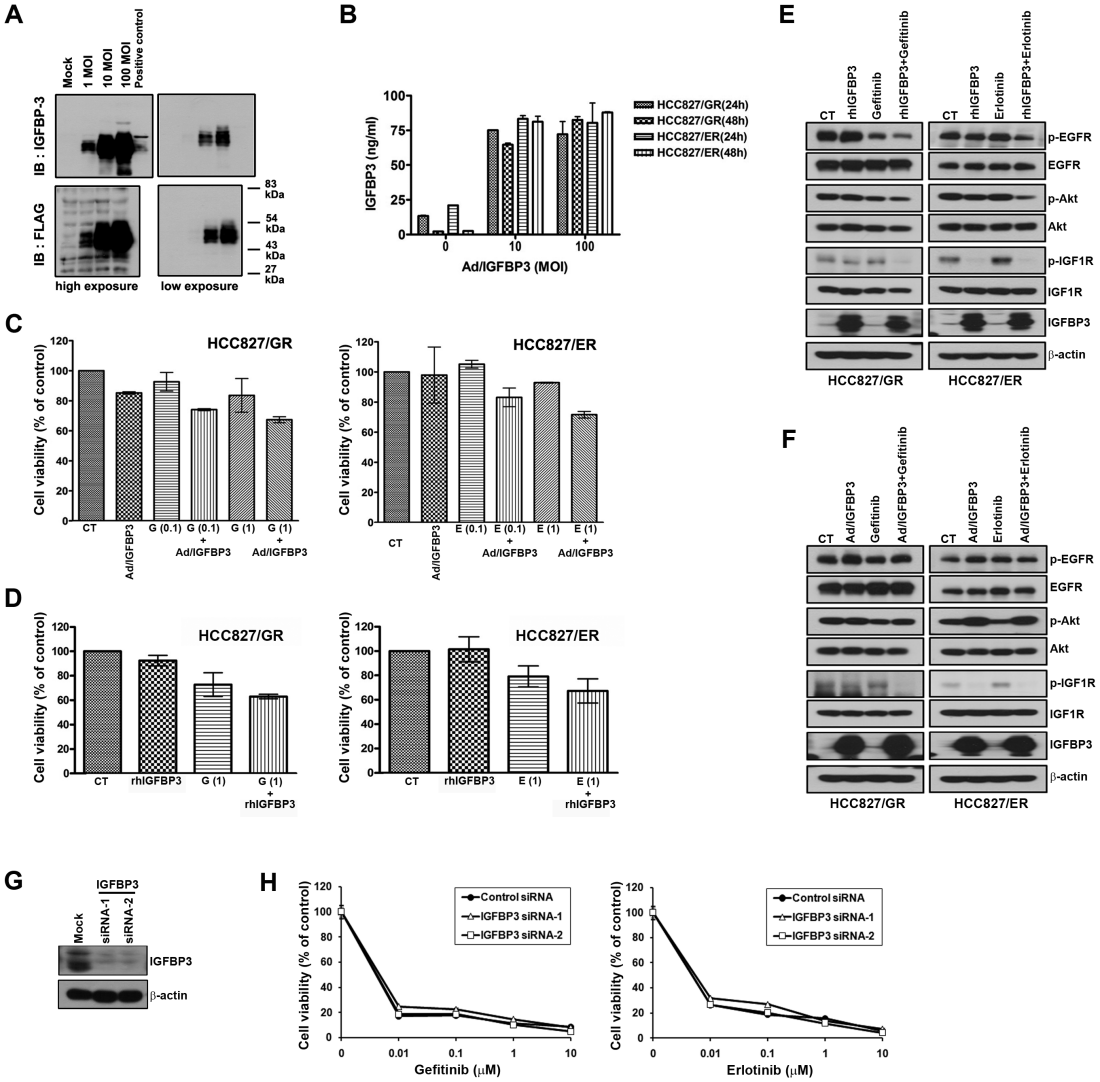
Effects of increased IGFBP3 on the sensitivity to EGFR-TKIs. Resistant cells were infected with Ad/IGFBP-3 at MOIs of 0 to 100 PFU/cell for 48 h and IGFBP-3 expression was determined by Western blotting (A) and ELISA (B). (C) HCC827/GR and HCC827/ER cells were treated with the indicated concentration of EGFR-TKIs for 72 h after infection with 100 MOI of Ad/IGFBP-3. (D) HCC827/GR and HCC827/ER cells were treated with 1 µM EGFR-TKIs and 1 µg/mL rh IGFBP-3 for 72 h. Results are representative of at least three independent experiments, and the error bars represent standard deviation (SD). (E and F) Cells were treated with drugs, rh IGFBP-3 or Ad/IGFBP-3 as in panel C and D. After 24 h, cells were harvested and the modulation of EGFR and IGF1R signalling in the indicated cell lines was detected by Western blotting. (G) Control and IGFBP-3 siRNAs (100 nM) were introduced into HCC827 cells, and IGFBP-3 suppression was confirmed by Western blotting. (H) Cell viability was measured using the MTT assay 72 h later.

To investigate the involvement of IGFBP-3 in the resistance to EGFR-TKIs, HCC827/GR cells were mock-infected or infected with Ad/IGFBP-3, and induction of IGFBP-3 expression was evaluated by Western blotting. As shown in Figure 4A, IGFBP-3 levels were significantly higher in HCC827/GR cells infected with various titres of ad/IGFBP-3 than in mock-infected cells, which was confirmed by Western blotting with an anti-flag antibody. Furthermore, IGFBP-3 secretion into the culture medium of infected HCC827/GR and HCC827/ER was increased as measured by ELISA (Figure 4B). To examine the effect of EGFR-TKIs on IGFBP-3 overexpressing cells, cells were infected with Ad/IGFBP-3 at 100 MOI and treated with EGFR-TKIs. However, co-treatment with Ad/IGFBP-3 and EGFR-TKIs did not effectively inhibit cell proliferation (Figure 4C). To examine the role of IGFBP-3 in resistance further, HCC827/GR and HCC827/ER cells were treated with 1 µg/mL recombinant human (rh) IGFBP-3 and 1 µM EGFR-TKIs for 72 h. Combined treatment resulted in modest growth inhibition in HCC827/GR and HCC827/ER (37.0% and 32.8%, respectively) despite the high concentration of rh IGFBP-3 (Figure 4D). Although the restoration of IGFBP-3 inhibited IGF1R activity, it could not reduce Akt activity (Figure 4E and F). Similarly, the suppression of IGFBP-3 slightly enhanced the IGF1R activity (data not shown), but sensitivity to EGFR-TKIs was not affected (Figure 4G and H). These results showed that although induction of IGFBP-3 slightly increased the effect of EGFR-TKIs, it was insufficient to overcome EGFR-TKI-acquired resistance, indicating that loss of IGFP-3 may not be the main resistance mechanism in HCC827 cells.

### Serum IGFBP3 Levels are not Correlated with Resistance to EGFR-TKIs

**Figure 5:**
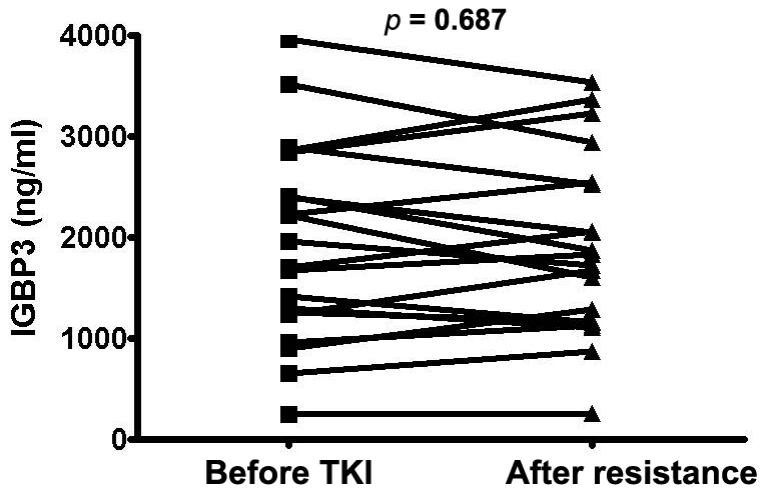
Serum IGFBP3 levels before EGFR-TKI treatment and after the development of resistance. IGFBP-3 levels were determined by ELISA in the serum of patients with NSCLC. Acquired resistance developed in all patients who initially responded to EGFR-TKIs.

Serum IGFBP-3 level was measured by ELISA in 20 NSCLC patients that showed partial responses to EGFR-TKI treatment. Of these 20 patients, 16 had EGFR mutations and four were not evaluated. Serum IGFBP-3 levels were not altered in response to the development of resistance to EGFR-TKIs (1815.3±94.6 ng/mL before treatment vs. 1778.9±87.8 ng/mL after EGFR-TKI resistance, p = 0.678, Figure 5).

## Discussion

The IGF pathway plays a role in the regulation of foetal development, tissue growth and metabolism. Two distinct ligands (IGF-1 and IGF-2) plus insulin, and two receptors (IGF-1R and the insulin receptor) capable of both homo- and heteropolymerisation mediate the actions of this pathway [22]. IGF-1R has a 15- to 20-fold higher affinity for IGF-1 than for IGF-2, and all the IGFBPs have a greater affinity for the IGF ligand than the corresponding IGF-receptors. Therefore, the activities of IGF are strictly regulated by a family of IGFBPs, and at least six IGFBPs (IGFBP-1 to IGFBP-6) have been identified. Among them, IGFBP-3 is the dominant circulating binding partner of IGF, accounting for 70−80% of IGF-1 binding [23]. IGFBP-3 has long been established as a potent negative regulator of IGF-1R activation and is believed to block ligand binding, although it may also have IGF-independent antiproliferative activities [24].

Deregulation of IGF signalling has been described in several cancer types, including lung cancer. Recently, inhibition of IGFR was shown to enhance the growth-inhibitory and apoptotic effects of geftinib in H1650 cells, which display primary resistance to EGFR-TKIs despite having a deletion mutation on exon 19 of the EGFR gene. This result suggested that combined inhibition of IGFR could be useful to overcome resistance to EGFR-TKIs in lung cancer [25]. A number of IGF receptor inhibitors, including monoclonal antibodies and small molecule inhibitors are currently in clinical trials.

IGFBP-3 expression is markedly downregulated in A431 gefitinib-resistant cells, which are developed under conditions of chronic EGFR inhibition [18], and treatment of these cells with AG1478, an EGFR-TKI, downregulates IGFBP-3 [26]. These results may explain the adaptation of cells grown under conditions of EGFR inhibition, which activate the IGFIR pathway leading to PI3K/AKT signalling. Re-exposure of A431 GR cells to IGFBP-3 resensitised both the PI3K pathway and cell survival to the effects of gefitinib [18]. However, a model of acquired resistance to gefitinib in EGFR mutant lung cancer cells has not been developed to date, despite the fact that the acquisition of EGFR-TKI resistance in NSCLC patients with EGFR mutations underscores its clinical relevance. Therefore, in the present study, we established two EGFR-TKI-resistant sublines (HCC827/GR and HCC827/ER) from parental HCC827 cells, which are sensitive to EGFR-TKIs because of the deletion mutation in exon 19, by continuous exposure to gefitinib and erlotinib.

Both cell lines showed downregulation of IGFBP-3 expression by Western blotting without changes in transcriptional activity and regardless of the resistance mechanism. Furthermore, the level of secreted IGFBP-3 in the culture medium of resistant cells was significantly reduced as shown by ELISA. However, no changes in IGFR signalling were detected despite the loss of IGFBP-3. This result contradicts that of a previous study showing that loss of expression of IGFBPs in tumour cells treated with EGFR-TKIs activates IGFIR signalling [18]. This discrepancy could have been caused by simultaneous changes in ligands such as IGF-1 or IGF-2, level of IGFBP-3 although this notion should be explored further.

Lee et al. examined the effects of IGFBP-3 on NSCLC cells after infection with an adenovirus constitutively expressing IGFBP-3 under the control of the cytomegalovirus promoter (Ad5CMV-BP3). IGFBP-3 overexpression inhibited the phosphorylation of Akt and glycogen synthase kinase-3β and the activity of MAPK. Furthermore, IGF-1 rescued the NSCLC cells from serum depletion-induced apoptosis, and this rescue was blocked in Ad5CMVBP-3-infected H1299 NSCLC cells [21]. However, in the present study, forced expression of IGFBP-3 by infection with an adenoviral vector or addition of recombinant IGFBP-3 only slightly increased the growth-inhibitory and apoptotic effects of EGFR-TKIs. A study by Chang et al. showed that decreased IGFBP-3 expression was significantly associated with shorter disease-specific survival in stage I NSCLC patients and indicated that hypermethylation of the IGFBP-3 promoter might be associated with the downregulation of IGFBP-3 [27], [28]. The importance of IGFBP-3 in the regulation of NSCLC cell proliferation, clonogenicity, and tumour growth was shown in an in vitro study by the same group. However, in a more recent study, IGFBP-3 expression was not correlated with any clinical variables and was not significantly associated with response to chemotherapy and survival [29], [30].

Overall, an association between EGFR-TKI treatment and changes in IGFBP-3 levels has not been supported by current data. In the present study, serum IGFBP-3 levels measured before EGFR-TKI treatment and after the development of EGFR-TKI resistance in 20 NSCLC patients showed no significant changes (1815.3±94.6 ng/mL before treatment vs. 1778.9±87.8 ng/mL after EGFR-TKI resistance). IGFBP-3 is mainly produced and released by hepatic Kupffer and endothelial cells into the systemic circulation to affect auxological growth via endocrine regulation [31]. Therefore, the proportion of circulating IGFBP-3 secreted from lung cancer cells may be too small for the detection of significant changes related to the development of resistance, despite the loss of IGFBP-3 in lung cancer cells.

Finally, we repeated same experiments on PC-9 cells with a deletion mutation on exon 19 of EGFR to validate our observation. Previously, we established gefitinib-resistant cells (PC-9/GR) from PC-9 cells [32]. Although PC-9/GR cells acquired T790M-mediated resistance, decreased expression of IGFBP-3 was also found in them (Supplementary Figure S1). Accordingly, reintroduction of IGFBP-3 in PC-9/GR cells or silencing of IGFBP-3 in PC-9 cells did not affect sensitivity to EGFR-TKIs.

In summary, although IGFBP-3 downregulation is associated with the acquisition of EGFR-TKI resistance regardless of the underlying mechanism, its modest effect on resistance detected in the present study suggests that IGFBP-3 does not play a major role in the resistance of NSCLC cells to EGFR-TKI therapy. Furthermore, our results suggest that serum IGFBP-3 is not a useful marker of resistance in advanced NSCLC.

## Supporting Information

Figure S1 **IGFBP-3 expression did not affect sensitivity to EGFR-TKIs in PC-9 cells.** Basal expression and mRNA of IGFBP-3 in PC-9, PC-9/GR and PC-9/ER cells were evaluated by Western blotting (A) and RT-PCR (B). (C) PC-9/GR cells were infected with Ad/IGFBP-3 at MOIs of 0 to 100 PFU/cells for 24 h and IGFBP-3 expression was determined by Western blotting. (D) PC-9/GR cells were treated with the indicated concentration of gefitinib and 1 µg/mL rh IGFBP-3 for 72 h after infection with 100 MOI of Ad/IGFBP-3. Cell viability was measured using an ADAM-MC automatic cell counter. Results are representative of at least three independent experiments, and the error bars represent standard deviation (SD). (E) Control and IGFBP-3 siRNA (100 nM) were introduced into PC-9 cells, and IGFBP-3 suppression was confirmed by Western blotting. (F) Cell viability was measured using the MTT assay 72 h later.
(TIF)

## References

[1] Salomon DS, Brandt R, Ciardiello F, Normanno N (1995) Epidermal growth factorrelated peptides and their receptors in human malignancies. Crit Rev Oncol Hematol 19: 183–232. 7612182 10.1016/1040-8428(94)00144-i

[2] Ciardiello F, Tortora G (2008) EGFR antagonists in cancer treatment. N Engl J Med 358: 1160–1174. 18337605 10.1056/NEJMra0707704

[3] Paez JG, Jänne PA, Lee JC, Tracy S, Greulich H, et al (2004) EGFR mutations in lung cancer: correlation with clinical response to gefitinib therapy. Science 304: 1497–1500. 15118125 10.1126/science.1099314

[4] Lynch TJ, Bell DW, Sordella R, Gurubhagavatula S, Okimoto RA, et al (2004) Activating mutations in the epidermal growth factor receptor underlying responsiveness of nonsmall-cell lung cancer to gefitinib. N Engl J Med 350: 2129–2139. 15118073 10.1056/NEJMoa040938

[5] Pao W, Miller V, Zakowski M, Doherty J, Politi K, et al (2004) EGF receptor gene mutations are common in lung cancers from “never smokers” and are associated with sensitivity of tumors to gefitinib and erlotinib. Proc Natl Acad Sci U S A 101: 13306–13311. 15329413 10.1073/pnas.0405220101 PMC516528

[6] Mitsudomi T, Kosaka T, Endoh H, Horio Y, Hida T, et al (2005) Mutations of the epidermal growth factor receptor gene predict prolonged survival after gefitinib treatment in patients with non-small-cell lung cancer with postoperative recurrence. J. Clin Oncol 23: 2513–2520. 15738541 10.1200/JCO.2005.00.992

[7] Han SW, Kim TY, Hwang PG, Jeong S, Kim J, et al (2005) Predictive and prognostic impact of epidermal growth factor receptor mutation in non-small-cell lung cancer patients treated with gefitinib. J. Clin Oncol 23: 2493–2501. 15710947 10.1200/JCO.2005.01.388

[8] Takano T, Ohe Y, Sakamoto H, Tsuta K, Matsuno Y, et al (2005) Epidermal growth factor receptor gene mutations and increased copy numbers predict gefitinib sensitivity in patients with recurrent non–small-cell lung cancer. J. Clin Oncol 23: 6829–6837. 15998907 10.1200/JCO.2005.01.0793

[9] Cappuzzo F, Hirsch FR, Rossi E, Bartolini S, Ceresoli GL, et al (2005) Epidermal growth factor receptor gene and protein and gefitinib sensitivity in non–small-cell lung cancer. J Natl Cancer Inst 97: 643–655. 15870435 10.1093/jnci/dji112

[10] Mitsudomi T, Yatabe Y (2007) Mutations of the epidermal growth factor receptor gene and related genes as determinants of epidermal growth factor receptor tyrosine kinase inhibitors sensitivity in lung cancer. Cancer Sci 98: 1817–1824. 17888036 10.1111/j.1349-7006.2007.00607.x PMC11159145

[11] Kobayashi S, Boggon TJ, Dayaram T, Jänne PA, Kocher O, et al (2005) EGFR mutation and resistance of non-small-cell lung cancer to gefitinib. N Engl J Med 352: 786–792. 15728811 10.1056/NEJMoa044238

[12] Pao W, Miller VA, Politi KA, Riely GJ, Somwar R, et al (2005) Acquired resistance of lung adenocarcinomas to gefitinib or erlotinib is associated with a second mutation in the EGFR kinase domain. PLoS Med 2: e73. 15737014 10.1371/journal.pmed.0020073 PMC549606

[13] Kosaka T, Yatabe Y, Endoh H, Yoshida K, Hida T, et al (2006) Analysis of epidermal growth factor receptor gene mutation in patients with non-small cell lung cancer and acquired resistance to gefitinib. Clin Cancer Res. 12: 5764–5769. 10.1158/1078-0432.CCR-06-0714 17020982

[14] Sequist LV, Waltman BA, Dias-Santagata D, Digumarthy S, Turke AB, et al (2011) Genotypic and histological evolution of lung cancers acquiring resistance to EGFR inhibitors. Sci Transl Med 3: 75ra26. 10.1126/scitranslmed.3002003 PMC3132801 21430269

[15] Engelman JA, Zejnullahu K, Mitsudomi T, Song Y, Hyland C, et al (2007) MET amplification leads to gefitinib resistance in lung cancer by activating ERBB3 signaling. Science 316: 1039–1043. 17463250 10.1126/science.1141478

[16] Bean J, Brennan C, Shih JY, Riely G, Viale A, et al (2007) MET amplification occurs with or without T790M mutations in EGFR mutant lung tumors with acquired resistance to gefitinib or erlotinib. Proc Natl Acad Sci U S A 104: 20932–20937. 18093943 10.1073/pnas.0710370104 PMC2409244

[17] Zhang Z, Lee JC, Lin L, Olivas V, Au V, et al (2012) Activation of the AXL kinase causes resistance to EGFR-targeted therapy in lung cancer. Nat Genet. 44: 852–860. 10.1038/ng.2330 PMC3408577 22751098

[18] Guix M, Faber AC, Wang SE, Olivares MG, Song Y, et al (2008) Acquired resistance to EGFR tyrosine kinase inhibitors in cancer cells is mediated by loss of IGF-binding proteins. J Clin Invest. 118: 2609–2619. 10.1172/JCI34588 PMC2430495 18568074

[19] Jones JI, Clemmons DR (1995) Insulin-like growth factors and their binding proteins: biological actions. Endocr Rev 16: 3–34. 7758431 10.1210/edrv-16-1-3

[20] Yu H, Spitz MR, Mistry J, Gu J, Hong WK, et al (1999) Plasma levels of insulin-like growth factor-I and lung cancer risk: a case-control analysis. J Natl Cancer Inst 91: 151–1516. 9923856 10.1093/jnci/91.2.151

[21] Lee HY, Chun KH, Liu B, Wiehle SA, Cristiano RJ, et al (2002) Insulin-like growth factor binding protein-3 inhibits the growth of non-small cell lung cancer. Cancer Res. 62: 3530–3537. 12068000

[22] Rosenzweig SA, Atreya HS (2010) Defining the pathway to insulin-like growth factor system targeting in cancer. Biochem Pharmacol 80: 1115–1124. 20599789 10.1016/j.bcp.2010.06.013 PMC2934757

[23] Dziadziuszko R, Camidge DR, Hirsch FR (2008) The insulin-like growth factor pathway in lung cancer. J Thorac Oncol 3: 815–818. 18670298 10.1097/JTO.0b013e31818180f5

[24] Grimberg A, Cohen P (2000) Role of insulin-like growth factors and their binding proteins in growth control and carcinogenesis. J Cell Physiol 183: 1–9. 10699960 10.1002/(SICI)1097-4652(200004)183:1<1::AID-JCP1>3.0.CO;2-J PMC4144680

[25] Choi YJ, Rho JK, Jeon BS, Choi SJ, Park SC, et al (2010) Combined inhibition of IGFR enhances the effects of gefitinib in H1650: a lung cancer cell line with EGFR mutation and primary resistance to EGFR-TK inhibitors. Cancer Chemother Pharmacol 66: 381–388. 19921194 10.1007/s00280-009-1174-7

[26] Takaoka M, Harada H, Andl CD, Oyama K, Naomoto Y, et al (2004) Epidermal growth factor receptor regulates aberrant expression of insulin-like growth factor-binding protein 3. Cancer Res 64: 7711–7723. 15520175 10.1158/0008-5472.CAN-04-0715 PMC4140096

[27] Chang YS, Kong G, Sun S, Liu D, El-Naggar AK, et al (2002) Clinical significance of insulin-like growth factorbinding protein-3 expression in stage I non-small cell lung cancer. Clin Cancer Res 8: 3796–3802. 12473592

[28] Chang YS, Wang L, Liu D, Mao L, Hong WK, et al (2002) Correlation between insulin-like growth factor binding protein-3 promoter methylation and prognosis of patients with stage I non-small cell lung cancer. Clin Cancer Res 8: 3669–3675. 12473575

[29] Kim YH, Sumiyoshi S, Hashimoto S, Masago K, Togashi Y, et al (2012) Expressions of Insulin-Like Growth Factor Receptor-1 and Insulin-Like Growth Factor Binding Protein 3 in Advanced Non-Small-Cell Lung Cancer. Clin Lung Cancer 13: 385–390. 22285568 10.1016/j.cllc.2011.11.009

[30] Masago K, Fujita S, Togashi Y, Kim YH, Hatachi Y, et al (2011) Clinical significance of epidermal growth factor receptor mutations and insulin-like growth factor 1 and its binding protein 3 in advanced non-squamous non-small cell lung cancer Oncol Rep. 26: 795–803. 10.3892/or.2011.1354 21805046

[31] Zimmermann EM, Li L, Hoyt EC, Pucilowska JB, Lichtman S, et al (2000) Cell-specific localization of insulin-like growth factor binding protein mRNAs in rat liver. Am J Physiol Gastrointest Liver Physiol 278: G447–G457. 10712265 10.1152/ajpgi.2000.278.3.G447

[32] Rho JK, Choi YJ, Lee JK, Ryoo BY, Na II, et al (2009) The role of MET activation in determining the sensitivity to epidermal growth factor receptor tyrosine kinase inhibitors. Mol Cancer Res 7: 1736–1743. 19808904 10.1158/1541-7786.MCR-08-0504

