# Correction: Role of IGF-Binding Protein 3 in the Resistance of EGFR Mutant Lung Cancer Cells to EGFR-Tyrosine Kinase Inhibitors

**DOI:** 10.1371/journal.pone.0213984

**Published:** 2019-03-14

**Authors:** Yun Jung Choi, Gun Min Park, Jin Kyung Rho, Sun Ye Kim, Gwang Sup So, Hyeong Ryul Kim, Chang-Min Choi, Jae Cheol Lee

There is an error in Fig 2B, due to duplication of the actin images in the HCC827/GR and HCC827/ER panels. Please see the correct Fig 2 here.

**Fig 2:**
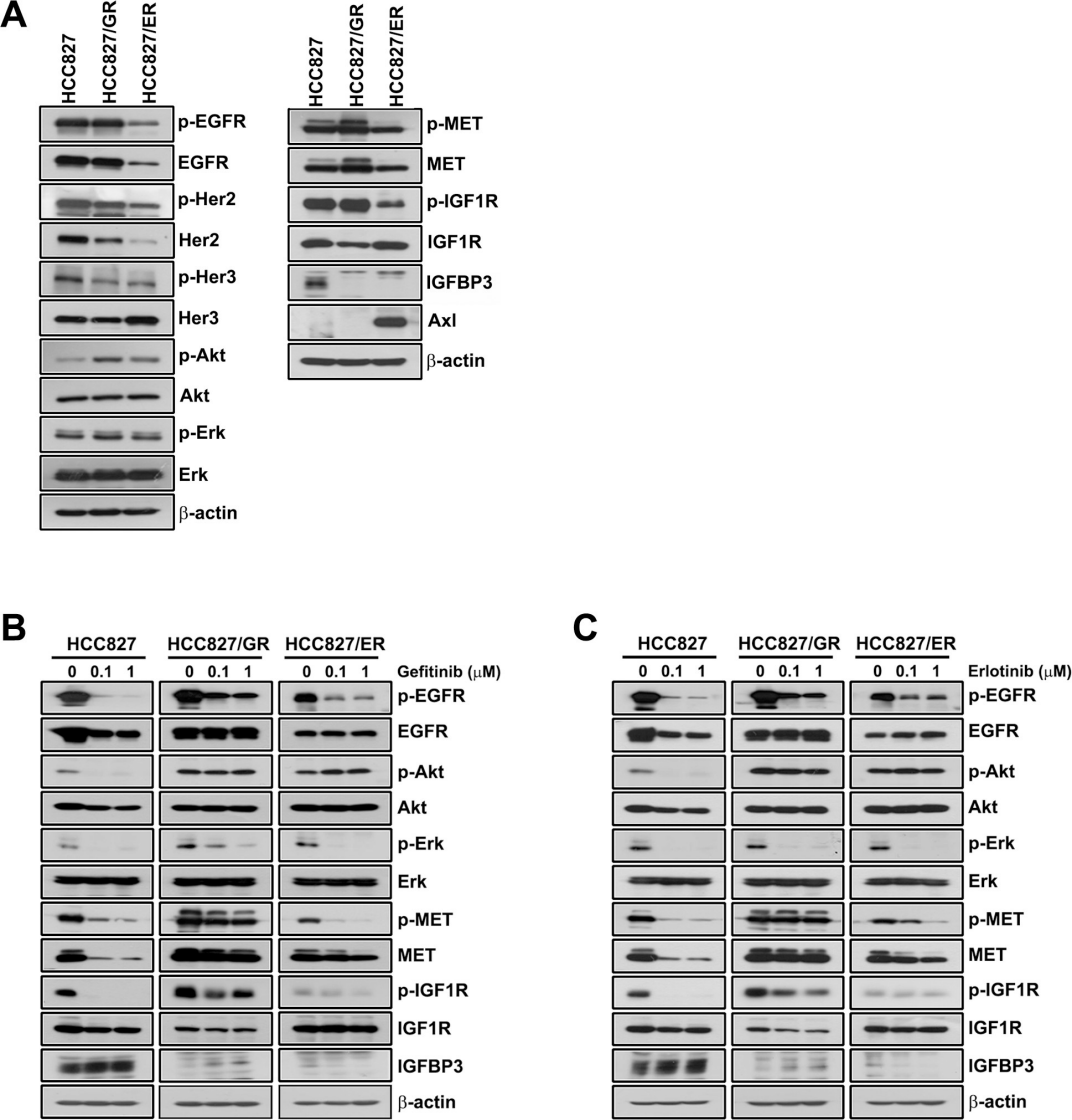
Expression of EGFR-related signals in HCC827 and resistant cell lines. (A) Basal expression of EGFR and EGFR-related signalling molecules in HCC827, HCC827/GR and HCC827/ER cells were evaluated by Western blotting. The effects of gefitinib (B) and erlotinib (C) on EGFR-related signalling were also examined. HCC827, HCC827/GR and HCC827/ER cells were treated with 0.1 and 1 μM gefitinib and erlotinib for 72 h in medium containing 1% FBS. Protein (30 μg) from cell lysates was subjected to Western blot analysis for the indicated proteins.

The unaltered, uncropped blot images underlying all Western blot figures in the article are provided as a Supporting Information file. It can be viewed below. Additional Western blot methodological information is provided by the authors as follows:

“We sliced blots horizontally using pre-stained markers and stained each separate blot with different primary antibodies. Also, we confirmed actin on the same gel accompanying target proteins. To validate phosphor-form and total form of proteins, western blot membranes were stripped and then re-probed according to protocol (Sigma-Aldrich). We put all figures in PowerPoint before we began making figures of the manuscripts in Photoshop. When we made the figures, we adjusted the blot images to have similar size. If any blot image was captured with larger size than others in PowerPoint, we adjusted the thickness and length of the protein bands to have similar size in Photoshop. However, we did not adjust the contrast and definition of the figures.”

## Supporting information

S1 File Western blots. Unaltered, uncropped blot images underlying all Western blot figures.
(PDF)
