# Supplementary figures and images for: Role of IGF-Binding Protein 3 in the Resistance of EGFR Mutant Lung Cancer Cells to EGFR-Tyrosine Kinase Inhibitors
Source: PLoS One. 2013 Dec 5;8(12):e81393. doi: 10.1371/journal.pone.0081393 (PMC3855319; doi:10.1371/journal.pone.0081393)

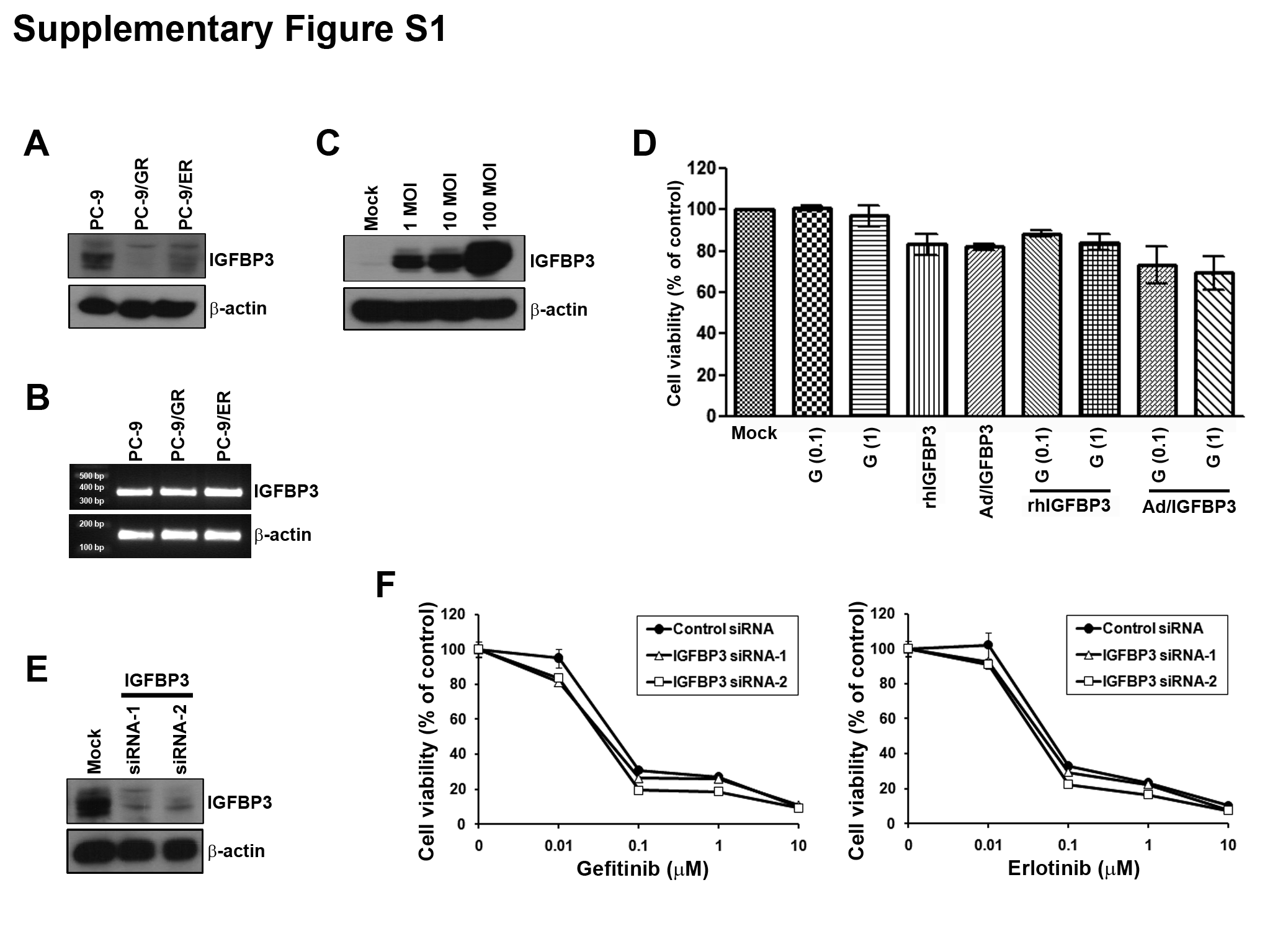

Supplement: Figure S1 — IGFBP-3 expression did not affect sensitivity to EGFR-TKIs in PC-9 cells. Basal expression and mRNA of IGFBP-3 in PC-9, PC-9/GR and PC-9/ER cells were evaluated by Western blotting (A) and RT-PCR (B). (C) PC-9/GR cells were infected with Ad/IGFBP-3 at MOIs of 0 to 100 PFU/cells for 24 h and IGFBP-3 expression was determined by Western blotting. (D) PC-9/GR cells were treated with the indicated concentration of gefitinib and 1 µg/mL rh IGFBP-3 for 72 h after infection with 100 MOI of Ad/IGFBP-3. Cell viability was measured using an ADAM-MC automatic cell counter. Results are representative of at least three independent experiments, and the error bars represent standard deviation (SD). (E) Control and IGFBP-3 siRNA (100 nM) were introduced into PC-9 cells, and IGFBP-3 suppression was confirmed by Western blotting. (F) Cell viability was measured using the MTT assay 72 h later. (TIF) [file pone.0081393.s001.tif]
