# Supplementary material for: Correction: Role of IGF-Binding Protein 3 in the Resistance of EGFR Mutant Lung Cancer Cells to EGFR-Tyrosine Kinase Inhibitors
Source: PLoS One. 2019 Mar 14;14(3):e0213984. doi: 10.1371/journal.pone.0213984 (PMC6417663; doi:10.1371/journal.pone.0213984)

OPEN ACCESS Freely available online
PLOS ONE

Role of IGF-Binding Protein 3 in the Resistance of EGFR Mutant Lung Cancer Cells to EGFR-Tyrosine Kinase Inhibitors

Yun Jung Choi[1]☯, Gun Min Park[5]☯, Jin Kyung Rho[1,2], Sun Ye Kim[1], Gwang Sup So[1], Hyeong Ryul Kim[3], Chang-Min Choi[1,4], Jae Cheol Lee[4]*

1 Department of Pulmonary and Critical Care Medicine, Asan Medical Center, College of Medicine, University of Ulsan, Seoul, Korea, **2** Asan Institute for Life Sciences, Asan Medical Center, College of Medicine, University of Ulsan, Seoul, Korea, **3** Department of Thoracic and Cardiovascular Surgery, Asan Medical Center, College of Medicine, University of Ulsan, Seoul, Korea, **4** Department of Oncology, Asan Medical Center, College of Medicine, University of Ulsan, Seoul, Korea, **5** Department of Internal Medicine, Daehan Hospital, Seoul, Korea

Abstract

Most patients treated with EGFR-tyrosine kinase inhibitors (EGFR-TKIs) eventually develop acquired resistance. Loss of expression of insulin-like growth factor (IGF)-binding protein-3 (IGFBP-3) has been suggested as a possible mechanism of resistance to EGFR-TKIs in the A431 and HN11 cell lines. Here, we investigated IGFBP-3 expression in two EGFR mutant lung cancer cell lines with resistance to EGFR-TKIs and examined the value of serum IGFBP-3 level as a marker of resistance. The effect of the induction or suppression of IGFBP-3 expression on resistance was also evaluated. HCC827 sublines with resistance to gefitinib (HCC827/GR) and erlotinib (HCC827/ER) were established. Loss of IGFBP-3 expression was detected by Western blotting in both cell lines without changes in transcriptional activity, and ELISA showed significantly lower amounts of secreted IGFBP-3 in the culture media of the mutant cell lines than in that of the parental line. Despite the loss of IGFBP-3 expression, IGFR signalling activity remained unchanged. Forced expression of IGFBP-3 by adenovirus-mediated transfection or recombinant IGFBP-3 slightly increased the growth-inhibitory and apoptotic effects of EGFR-TKIs, whereas suppression of IGFBP-3 did not affect sensitivity to EGFR-TKI. Serum IGFBP-3 levels measured by ELISA before and after the development of EGFR-TKI resistance in 20 patients showed no significant changes (1815.3 ± 94.6 ng/mL before treatment vs. 1778.9 ± 87.8 ng/mL after EGFR-TKI resistance). In summary, although IGFBP-3 downregulation is associated with the acquisition of resistance to EGFR-TKIs regardless of the mechanism, its effect on resistance was not significant, indicating that IGFBP-3 may not play an important role in resistance to EGFR-TKIs and serum IGFBP-3 level is not a reliable indicator of resistance.

Citation: Choi YJ, Park GM, Rho JK, Kim SY, So GS, et al. (2013) Role of IGF-Binding Protein 3 in the Resistance of EGFR Mutant Lung Cancer Cells to EGFR-Tyrosine Kinase Inhibitors. PLoS ONE 8(12): e81393. doi:10.1371/journal.pone.0081393

Editor: Giuseppe Viglietto, University Magna Graecia, Italy

Received May 13, 2013; **Accepted** October 14, 2013; **Published** December 5, 2013

Copyright: © 2013 Choi et al. This is an open-access article distributed under the terms of the Creative Commons Attribution License, which permits unrestricted use, distribution, and reproduction in any medium, provided the original author and source are credited.

Funding: This study was supported by a grant (2011-467) from Asan Institute for Life Science, Seoul, Korea. The funders had no role in study design, data collection and analysis, decision to publish, or preparation of the manuscript.

Competing Interests: The authors have declared that no competing interests exist.

* E-mail: jclee@ amc.seoul.kr

☯ These authors contributed equally to this work.

Introduction

EGFR is a transmembrane receptor that belongs to a family of four related proteins, EGFR (ErbB-1), HER2/neu (ErbB-2), HER3 (ErbB-3) and HER4 (ErbB-4) [1]. Upon ligand binding, EGFR forms homo- or heterodimers with other ErbB receptors leading to the activation of intracellular signalling cascades. The two major intracellular pathways activated by EGFR are the RAS-RAF-MEK-MAPK pathway, which controls gene transcription, cell-cycle progression and cell proliferation, and the PI3K-Akt pathway, which activates a cascade of anti-apoptotic and prosurvival signals [2].

Non-small cell lung cancers (NSCLCs) that harbour activating mutations and/or amplification of the EGFR locus are particularly sensitive to EGFR-tyrosine kinase inhibitors (TKIs) such as gefitinib (Iressa; AstraZeneca International) and erlotinib (Tarceva; OSI Pharmaceuticals) [3–9]. Approximately 70–80% of NSCLCs harbouring a somatic mutation in the tyrosine kinase domain of the EGFR gene respond to gefitinib/erlotinib [3,4,10]. However, acquired resistance to EGFR-TKI therapy almost always develops after a median of approximately 10 months from the onset of treatment, even in patients who exhibit an initial dramatic response to these agents. Acquired resistance has been associated with a secondary mutation in the EGFR gene, T790M [11,12], which has been detected in approximately 50% of cancers with acquired resistance to EGFR-TKIs [13,14]. In addition, amplification of the MET oncogene was identified as another mechanism of acquired resistance mediated by the phosphorylation of ErbB-3 and the consequent activation of PI3K [15,16]. Similarly, overexpression of the AXL kinase has been associated with resistance to EGFR-TKIs [17].

In a recent study, loss of expression of insulin-like growth factor (IGF)-binding protein 3 (IGFBP-3) was suggested as a possible mechanism of resistance in the A431 and HN11 cell lines [18]. In

Figure 2A

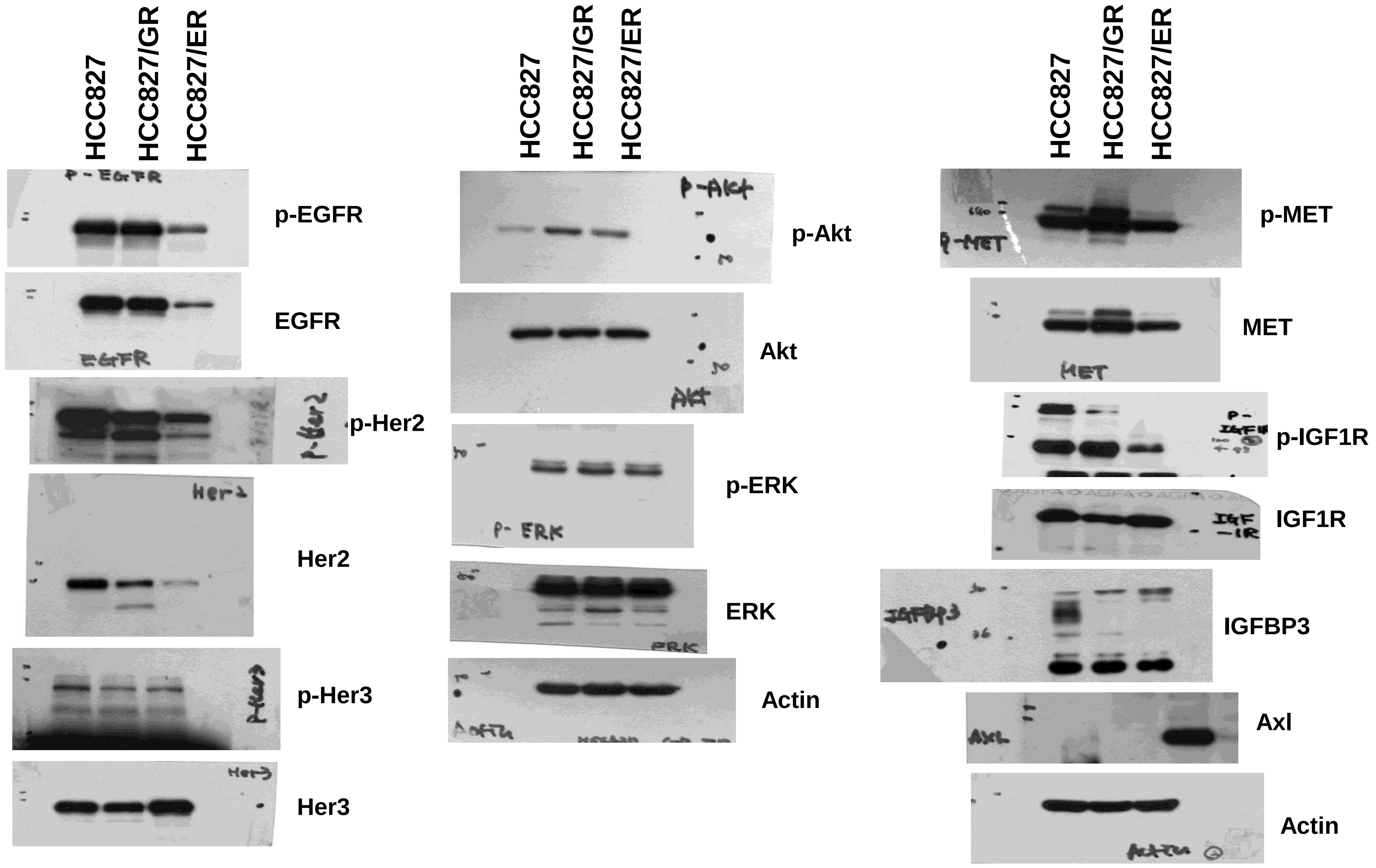
HCC827
HCC827/GR
HCC827/ER
p-EGFR
EGFR
p-Her2
Her2
p-Her3
Her3
p-Akt
Akt
p-ERK
ERK
Actin
p-MET
MET
p-IGF1R
IGF1R
IGFBP3
Axl
Actin

Figure 2B

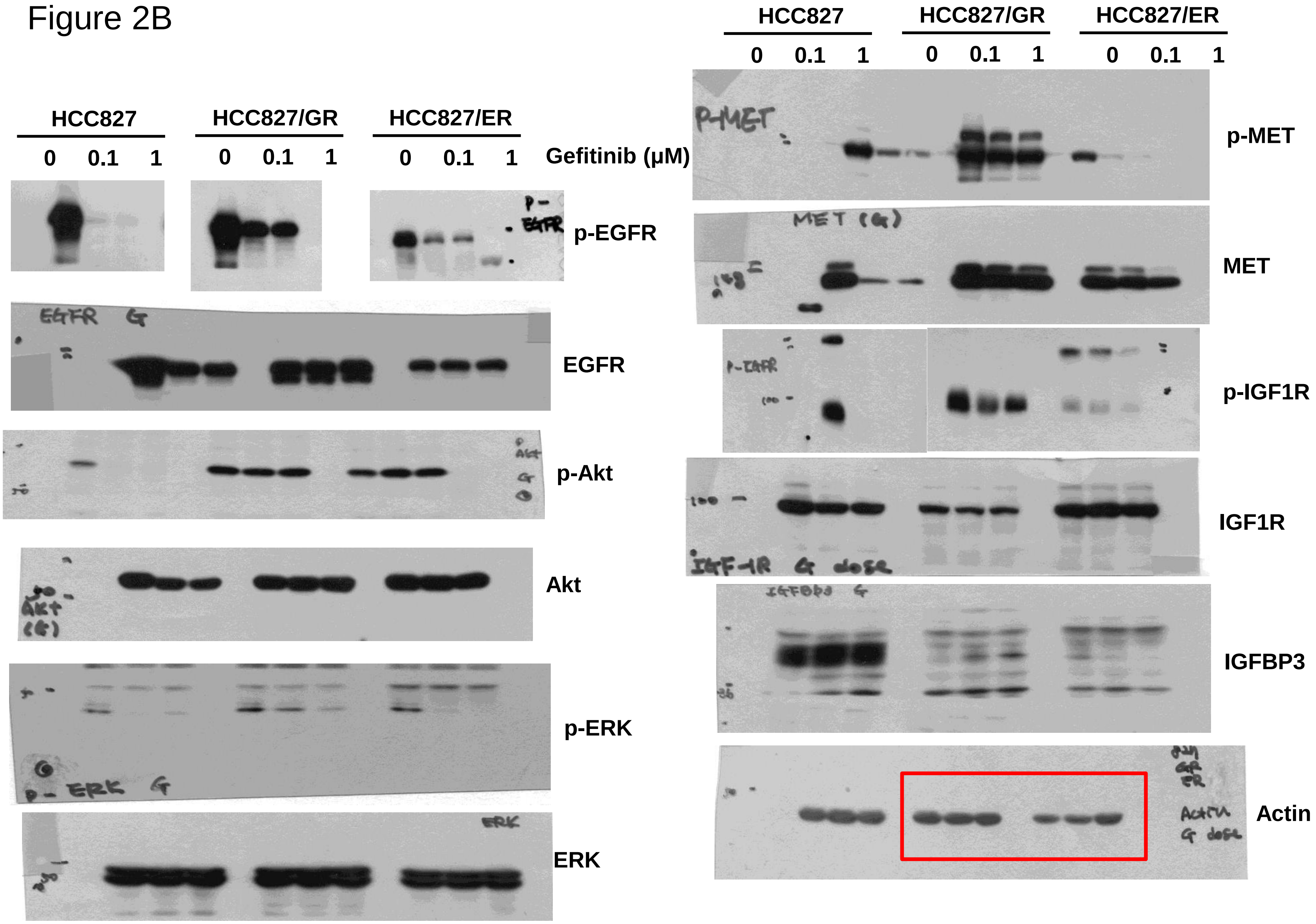
HCC827
HCC827/GR
HCC827/ER
0 0.1 1
Gefitinib (μM)
p-EGFR
EGFR
p-Akt
Akt
p-ERK
ERK
p-MET
MET
p-IGF1R
IGF1R
IGFBP3
Actin

Figure 2C

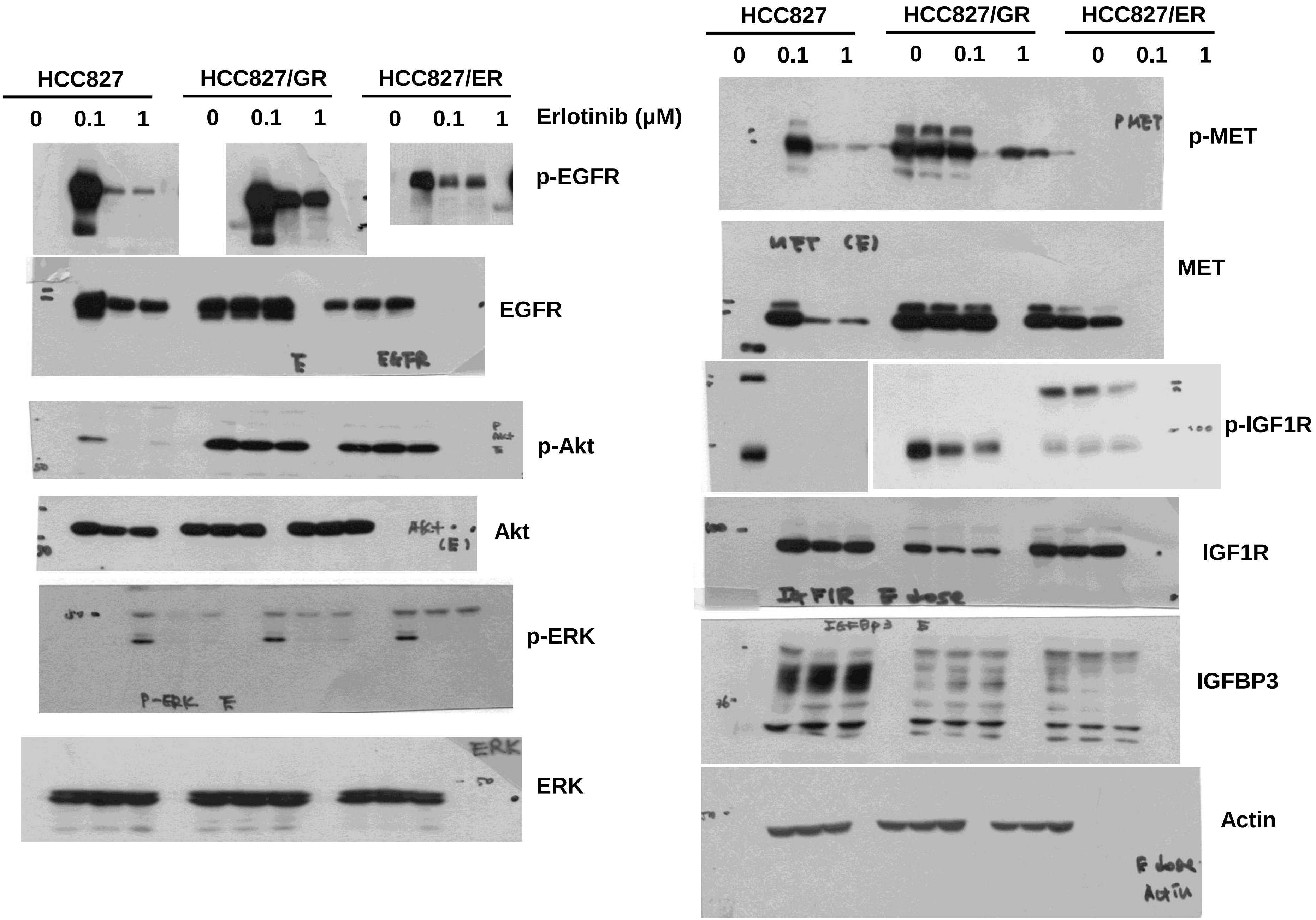
HCC827
HCC827/GR
HCC827/ER
0 0.1 1 0 0.1 1 0 0.1 1
Erlotinib (μM)
p-EGFR
EGFR
p-Akt
Akt
p-ERK
ERK
HCC827
HCC827/GR
HCC827/ER
0 0.1 1 0 0.1 1 0 0.1 1
p-MET
MET
p-IGF1R
IGF1R
IGFBP3
Actin

Figure 3A

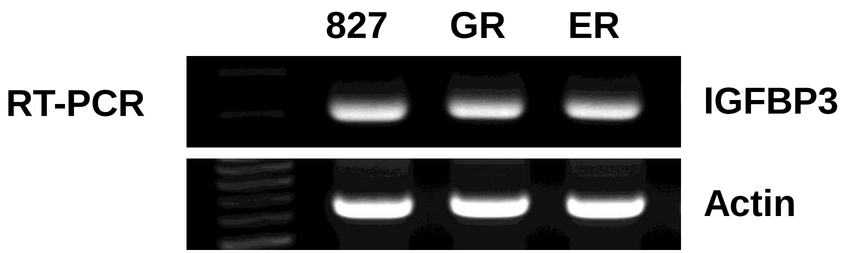

Figure 4A

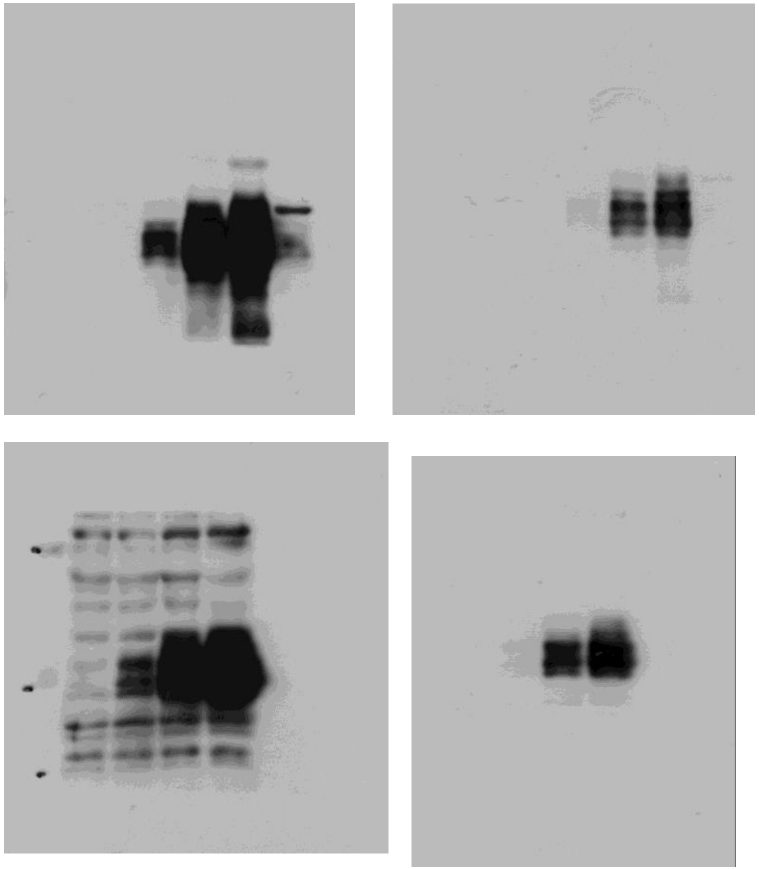

Figure 4E

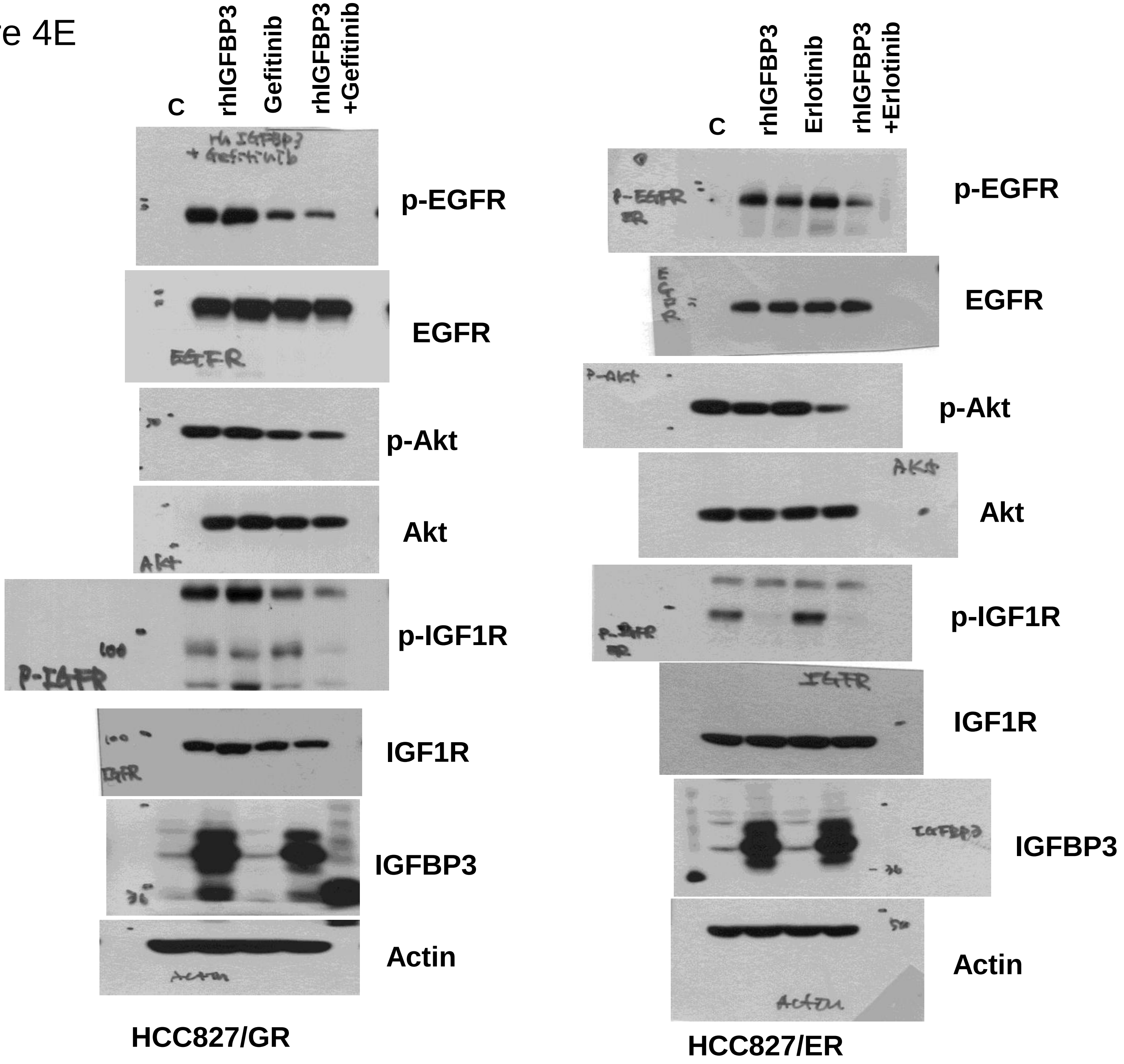
C
rhIGFBP3
Gefitinib
rhIGFBP3 +Gefitinib
p-EGFR
EGFR
p-Akt
Akt
p-IGF1R
IGF1R
IGFBP3
Actin
HCC827/GR
C
rhIGFBP3
Erlotinib
rhIGFBP3 +Erlotinib
p-EGFR
EGFR
p-Akt
Akt
p-IGF1R
IGF1R
IGFBP3
Actin
HCC827/ER

Figure 4F

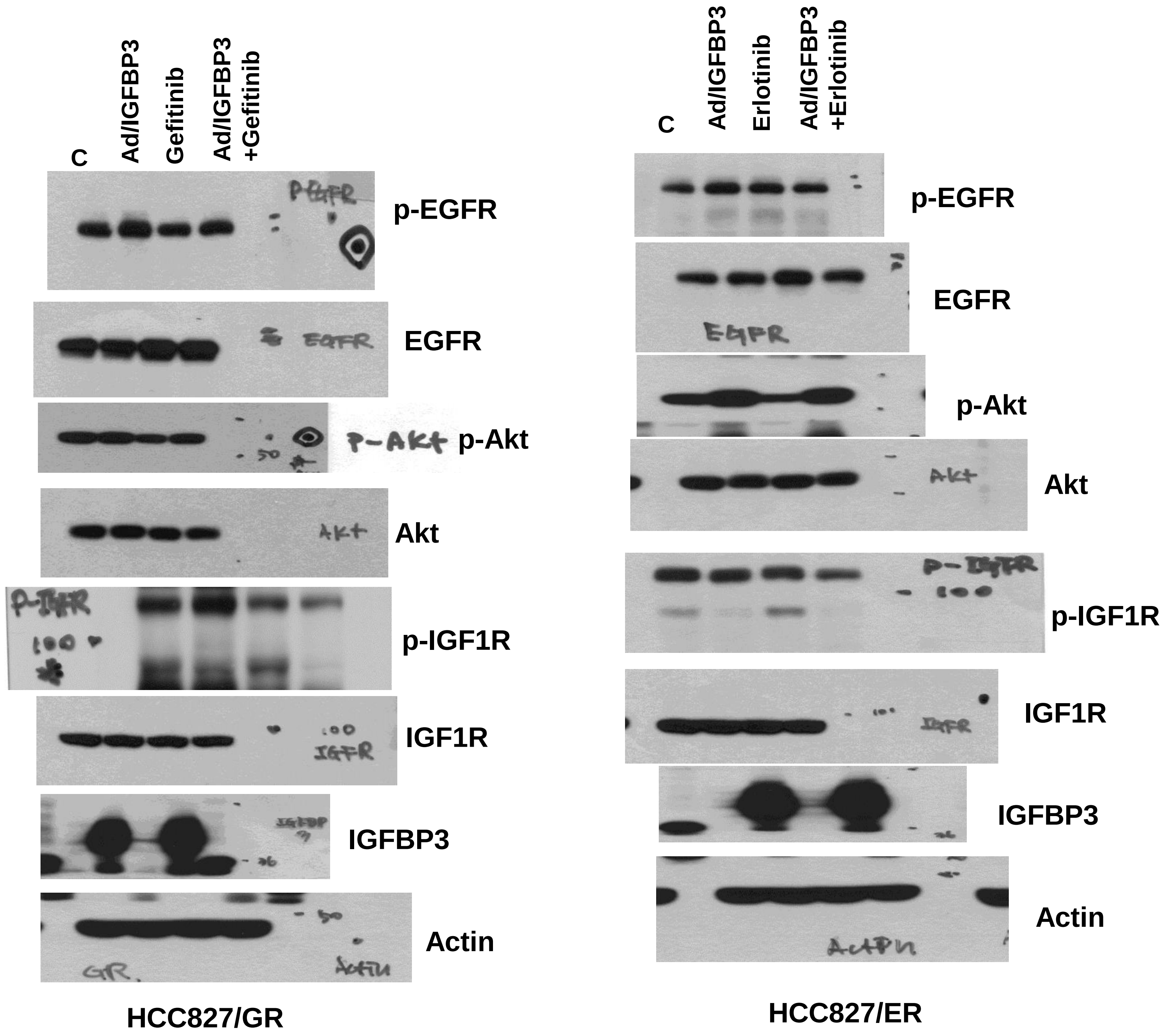
C
Ad/IGFBP3
Gefitinib
Ad/IGFBP3 +Gefitinib
p-EGFR
EGFR
p-Akt
Akt
p-IGF1R
IGF1R
IGFBP3
Actin
HCC827/GR
C
Ad/IGFBP3
Erlotinib
Ad/IGFBP3 +Erlotinib
p-EGFR
EGFR
p-Akt
Akt
p-IGF1R
IGF1R
IGFBP3
Actin
HCC827/ER

Figure 4G

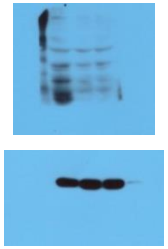

Supplement: S1 File — Unaltered, uncropped blot images underlying all Western blot figures. (PDF) [file pone.0213984.s001.pdf]
